# Antiurolithiatic Activity of Whole-Plant Hydroalcoholic Extract of *Pergularia daemia* in Rats

**DOI:** 10.4103/0975-1483.76417

**Published:** 2011

**Authors:** BA Vyas, RB Vyas, SV Joshi, DD Santani

**Affiliations:** *Maliba Pharmacy College, Gopal Vidyanagar, Tarsadi, Gujarat – 394 350, India*; 1 *Rofel, Shri G. M. Bilakhia College of Pharmacy, Vapi, India*

**Keywords:** Diuretic activity, ethylene glycol, hyperoxaluria, Lipid peroxidation, urolithiasis

## Abstract

The whole-plant, *Pergularia daemia* (Family: Asclepediaceae), extract (50% alcohol) was investigated for its antiurolithiatic and diuretic activity. Ethylene glycol (0.75% in water) feeding resulted in hyperoxaluria as well as increased renal excretion of calcium and phosphate. Alcoholic extract (400 mg/kg) of *P. daemia* was given orally in curative and preventive regimens over a period of 28 days. Supplementation with extract significantly (*P* < 0.001) lowered the urinary excretion and kidney retention levels of oxalate, calcium and phosphate. Furthermore, high serum levels of urea nitrogen, creatinine and uric acid were significantly (*P* < 0.001) reduced by the extract. The results were comparable with the standard drug, cystone (750 mg/kg). The reduction of stone-forming constituents in urine and their decreased kidney retention reduces the solubility product of crystallizing salts such as calcium oxalate and calcium phosphate, which could contribute to the antiurolithiatic property of the extract. The extract exhibited significant diuretic activity at dose of 400 mg/kg body weight as evidenced by increased total urine volume and the urine concentration of Na^+^, and K^+^. These findings affirm assertions made regarding the effectiveness of the extract of this plant against urinary pathologies in the Indian folk medicine.

## INTRODUCTION

Nephrolithiasis or renal stone disease remains a significant health problem in the adult population, with serious medical consequences, throughout a patient’s lifetime. The worldwide incidence of urolithiasis is quite high, and more than 80% of urinary calculi are calcium oxalate stones alone or calcium oxalate mixed with calcium phosphate.[1] The present-day medical management of nephrolithiasis is either costly or not without side-effects. Invasive procedures for the treatment of nephrolithiasis may cause serious complications and also impose a great load of costs on the healthcare system.

In contrast, traditional medicines have offered a substitute for many diseases and also have provided some supplementary information about the pathogenesis of diseases. *Pergularia daemia* Forsk. Syn. *Pergularia extensa* (Family: Asclepiadaceae), known as “Uttaravaruni” in Sanskrit, is a perennial herb growing widely along the roadsides of India. Traditionally, the plant is useful as an anthelmintic, laxative, anti-pyretic, expectorant, and used in infantile diarrhea.[2] Phytochemically the plant has been investigated for cardenolides, alkaloids, triterpenes and saponins.[3] The plant has been documented for anti-inflammatory, anti-pyretic and analgesic activities,[3] antifertility,[4] antidiabetic,[5] wound healing,[6] antibacterial,[7] and hepatoprotective activity.[8] Aanjaneyulu *et al*., 1998,[9] reported the presence of various triterpenes and steroidal compounds. The objective of the present study was to investigate and to validate the antiurolithiatic property of *Pergularia daemia* extract in experimentally-induced urolithiasis in rats.

## MATERIALS AND METHODS

### Plant material and extraction procedure

Plant *Pergularia daemia* was purchased from Botanical Survey of India, Jodhpur and authenticated at the Pharmacognosy dept. The plant material (500 g) was extracted with 50% alcohol using a Soxhlet apparatus. The extract was concentrated in a rotary evaporator at reduced pressure (yield 9.4% w/w).

### Phytochemical analysis

The extract was screened for various constituents (alkaloids, saponins, tannins, anthraquinones, sterol, flavonoids, terpenoids, glycosides, simple sugars) using standard protocol.

### Animals

Wistar rats (200-250 g) and Swiss albino mice of either sex were used. Animals were housed in groups of five under standard laboratory conditions of temperature (25 ± 2°C) and 12/12 h light/dark cycle. They were provided with standard pellets and tap water *ad libitum*. The study protocol was approved by the Institutional Animal Ethical Committee (IAEC) of CPCSEA (Committee for the Purpose of Control and Supervision of Experiments on Animals).

### Acute toxicity studies

Acute oral toxicity study was performed as per OECD-423 guidelines.[10] Albino mice (n = 6) of either sex selected by random sampling technique were used for acute toxicity study. The animals were kept fasting overnight providing only water, after which the extract (50% alcoholic extract) was administered orally at the dose level of 5 mg/kg body weight by gastric intubation and observed for 14 days. If mortality was observed in two out of three animals, then the dose administered was assigned as toxic dose. If mortality was observed in one animal, then the same dose would be repeated again to confirm the toxic dose. If mortality was not observed, the procedure was repeated for further higher doses such as 50, 300 and 2000 mg/kg body weight. According to the results of the acute toxicity test, the doses were chosen for experiments.

### Ethylene glycol-induced urolithiasis model[11]

Animals were divided into five different groups containing six animals each. Ethylene glycol (0.75% v/v) in drinking water was fed to all groups except control for induction of renal calculi till the 28^th^ day. Two types of study were performed, viz. curative study and preventive study. In the curative study all the groups except control received extract (400 mg/kg, once daily by oral route) from 15^th^ day till 28^th^ day[1] while in the preventive study all groups except control received extract (400 mg/kg, once daily by oral route) from 1^st^ day till 28^th^ day. Cystone was used as standard drug (750 mg/kg body weight). During the study animals were allowed free access to food.

Group – I: Control
Group – II: Ethylene glycol (0.75%) in drinking water + Vehicle
Group – III: Ethylene glycol (0.75%) in drinking water + Cystone (750 mg/kg)
Group – IV: Ethylene glycol (0.75%) in drinking water + extract (400 mg/kg) – Curative study
Group – V: Ethylene glycol (0.75%) in drinking water + extract (400 mg/kg) – Preventive study

### Assessment of antiurolithiatic activity

#### Collection and analysis of urine

All animals were kept in individual metabolic cages and urine samples of 24-h were collected on the 28^th^ day. Animals had free access to drinking water during the urine collection period. Urine was analyzed for calcium, phosphate and oxalate content using an automated system.

#### Serum analysis

Blood was collected from the retro-orbital under anesthetic conditions on the 28^th^ day. Serum was separated by centrifugation at 10,000 × g for 10 min and was analyzed for creatinine, urea nitrogen and uric acid using Automated Clinical Chemistry Analysis System.

#### Kidney homogenate analysis

Animals were sacrificed by cervical decapitation after the experimental period. The abdomen was cut open to remove both kidneys from each animal. The calcium, phosphate and oxalate content in the kidney homogenate were determined using an automated system. Further, kidney homogenate was used to study lipid peroxidation using the thiobarbituric acid reactive method.[12]

#### Diuretic activity

For the evaluation of diuretic activity, male albino rats weighing 200–250 g were selected. The control rats received 25 ml/kg saline by gastric gavage, while the treated groups received the same dose of normal saline containing 400 mg/10 ml extract. Furosemide (100 mg/kg) in 10 ml normal saline orally served as reference. Rats were kept separately in metabolic cages and had free access to water but not to food. The urine volume, electrolyte and pH were measured at 24 h.

### Statistical analysis

The data were expressed as mean ± SEM. Statistical significance between means was analyzed by one-way analysis of variance (ANOVA) followed by “Dunnett’s test.” *P* value <0.05 was considered statistically significant.

## RESULTS AND DISCUSSION

Result of preliminary phytochemical analysis conducted on 50% alcohol extract of *Pergularia daemia* showed presence of flavonoids, steroids, alkaloids and triterpens. Acute toxicity studies showed that the alcoholic extracts did not cause any mortality up to 2000 mg/kg and were considered as safe.[13]

Several *in vivo* models have been developed for the study of nephrolithiasis and to investigate the mechanisms involved in the formation of urinary stones. Rat has been a suitable species for study of CaOx deposition in the kidneys, a process that mimics the etiology of kidney stone formation in humans.[14] Several methods were used for induction of urolithiasis, which cause predominantly two types of hyperoxaluria, one acute, when the rat is challenged by a large single dose of lithogen and secondly chronic, when the rat is continuously challenged by small doses of lithogen for a period of time.

**Figure 1:**
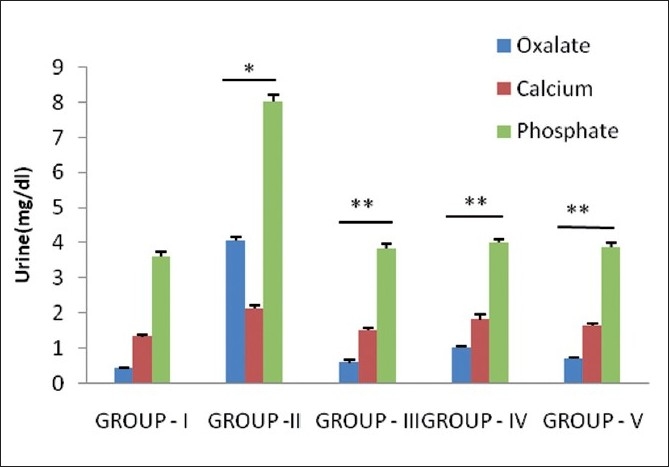
Effect of *P. daemia* extract on urinary parameters in control and experimental animals (All values represent mean ± S.E.M, N = 6, ^*^ *P* < 0.001, compared to control Group I, N = 6, ^**^ *P* < 0.001, compared to ethylene glycol Group II, One-way analysis of variance (ANOVA) followed by “Dunnett’s test.”)

In the present study, ethylene glycol-induced hyperoxaluria model[11] was used to assess the antilithiatic activity in albino rats. Chronic administration of 0.75% (v/v) ethylene glycol aqueous solution to male albino rats resulted in hyperoxaluria. Oxalate, calcium and phosphate excretion were increased in the calculi-induced (Group II) animals [Figure 1]. The biochemical mechanism involved in this process is associated with a raise in the urinary concentration of oxalate. Stone formation in ethylene glycol-fed animals is caused by hyperoxaluria, which causes increased renal retention and excretion of oxalate.[15]

**Figure 2:**
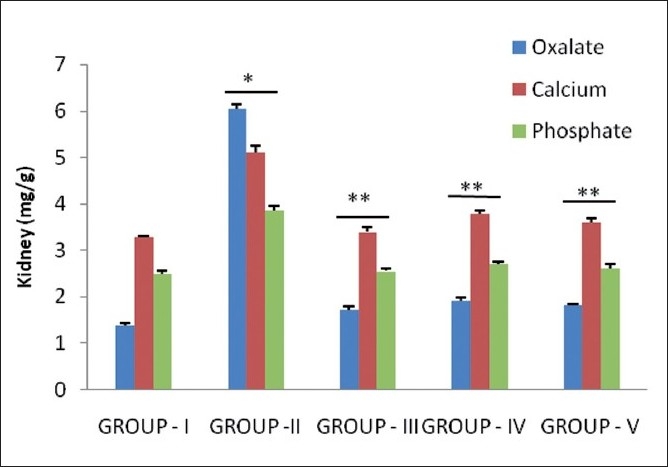
Effect of *P. daemia* extract on kidney parameters in control and experimental animals (All values represent mean ± S.E.M, N = 6, ^*^ *P* < 0.001, compared to control Group I, N = 6, ^**^ *P* < 0.001, compared to ethylene glycol Group II, One-way analysis of variance (ANOVA) followed by “Dunnett’s test.”)

Hyperoxaluria and hypercalciuria are major risk factors for the pathogenesis of renal stones. Since hyperoxaluria is a far more significant risk factor, the changes in urinary oxalate levels are comparatively much more imperative than those of calcium.[16] Increased urinary and kidney calcium is a factor stimulating the nucleation and precipitation of calcium oxalate or apatite (calcium phosphate) from urine and following crystal growth.[17] However, in the present study, supplementation with *Pergularia daemia* extract and Cystone restored oxalate and calcium in urine and kidney in curative regimens and preventive regimens as compared to calculi-treated animals [Figures 1 and 2].

Increased urinary phosphate excretion along with oxalate seems to provide an environment suitable for stone formation by forming calcium phosphate crystals, which epitaxially induce calcium oxalate deposition.[18] Treatment with *Pergularia daemia* extract significantly (*P*<0.001) lowered the elevated levels of phosphate in both the regimens, consequently reducing the menace of calculi formation.

**Figure 3:**
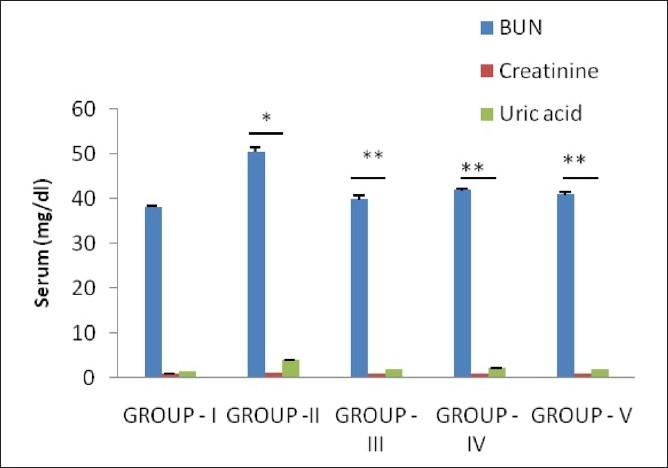
Effect of *P. daemia* extract on Serum parameters in control and experimental animals (All values represent mean ± S.E.M, N = 6, ^*^ *P* < 0.001, compared to control Group I, N = 6, ^**^ *P* < 0.001, compared to ethylene glycol Group II, One-way analysis of variance (ANOVA) followed by “Dunnett’s test.”)

The glomerular filtration rate (GFR) is an important parameter for ensuring renal function and it gets decreased in urolithiasis due to the obstruction to the outflow of the urine by stones in urinary system, which leads to a rise in nitrogenous waste products like urea, creatinine, and uric acid in blood.[19] In calculi-induced rats (Group II), marked renal damage was seen by the elevated serum levels of creatinine, uric acid and Blood Urea Nitrogen (BUN). However, the curative and prophylactic treatment with test extract restored the elevated serum levels of creatinine, uric acid and BUN [Figure 3].

Increased lipid peroxidation and decreased levels of antioxidant potential have been reported in the kidneys of rats supplemented with ethylene glycol.[20,21] Oxalate has been reported to induce lipid peroxidation and to cause renal tissue damage by reacting with polyunsaturated fatty acids in the cell membrane.[22]

**Figure 4:**
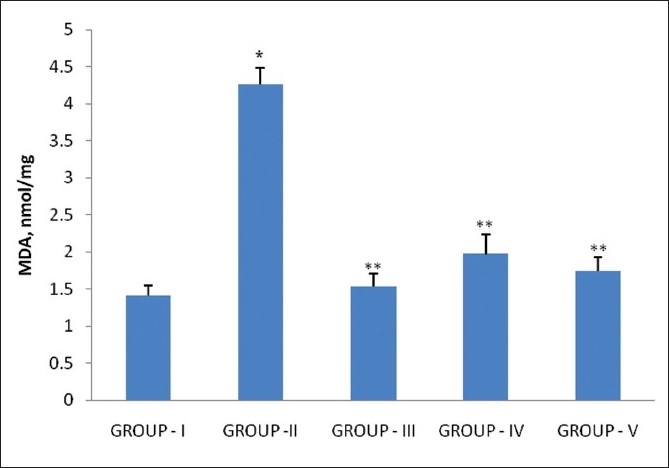
Effect of *P. daemia* extract on lipid peroxidation of kidney in control and experimental animals (All values represent mean ± S.E.M, N = 6, ^*^ *P* < 0.001, compared to control Group I, N = 6, ^**^ *P* < 0.001, compared to ethylene glycol Group II, One-way analysis of variance (ANOVA) followed by “Dunnett’s test.”)

In the present study, calculi-treated rats (Group II) showed an increase in malondialdehyde (MDA) content, representing lipid peroxidation, compared with untreated group in kidney. Administration of extract (400 mg/kg) and cystone (750 mg/kg) showed significant inhibition of lipid peroxide level in rat kidney in both the prophylactic and therapeutic groups [Figure 4]. Recent studies show increased urinary excretion of MDA in human calcium-oxalate kidney-stone-formers.[23] In addition, another recent study provided more indication signifying that oxidative stress plays a role in kidney-stone formation in humans.[24] The present results suggest that supplementation with *P. daemia* extract significantly decreased oxalate-induced kidney lipid peroxidation.

*P. daemia* extract with 400 mg/kg body weight exhibited significant (*P* <0.01) diuretic activity. The test extract significantly (*P*<0.01) increased the urine volume and concentration of Na^+^, and K^+^ in urine without any alteration in the Na^+^/K^+^ ratio when compared to the control group. Standard drug furosemide also exhibited significant activity [Table 1]. The results indicated that diuretic potential of the extract hastens the process of dissolving the preformed stones and prevention of new stone formation in the urinary system.

**Table 1:** Effect of *Pergularia daemia* extract on urine volume, electrolyte excretion and pH in the rats

Treatment	Urine volume ml/24 h	Electrolyte Concentration (meq/l)			pH
		Na^+^	K^+^	Na^+^/K^+^ ratio	
Normal Saline (25 ml/kg)	5.12 ± 1.51	84.52±0.48	55.71±1.23	1.51	7.1 ± 0.2
Furosemide (100 mg/kg)	9.28 ± 0.98*	139.11±0.61*	89.42±0.93*	1.55	6.3 ± 0.12
Alcoholic extract (400 mg/kg)	7.62 ± 1.22*	138.22±1.05*	82.93±0.82*	1.59	6.4 ± 0.16

Each value represents the mean ± S.E.M. of six rats,

* *P* < 0.001, Compared to normal saline-treated group, One-way analysis of variance (ANOVA) followed by “Dunnett’s test.”

In conclusion, the present findings affirm the assertions made regarding the effectiveness of the extract of this plant against urinary pathologies in the Indian folk medicine, i.e. as an antiurolithiatic drug. The mechanism underlying this effect is still unknown, but is apparently related to its diuretic properties and lowering of urinary concentrations of stone-forming constituents, which may be attributed to the presence of alkaloids, triterpenoids and flavonoids.
